# Identification of AKR1B10 as a key gene in primary biliary cholangitis by integrated bioinformatics analysis and experimental validation

**DOI:** 10.3389/fmolb.2023.1124956

**Published:** 2023-02-09

**Authors:** Huiwen Wang, Jian Zhang, Jinqing Liu, Yongfang Jiang, Lei Fu, Shifang Peng

**Affiliations:** ^1^ Department of Infectious Diseases, Xiangya Hospital, Central South University, Changsha, China; ^2^ Department of Infectious Diseases, Second Xiangya Hospital, Central South University, Changsha, China

**Keywords:** AKR1B10, primary biliary cholangitis, integrated bioinformatics, differentially expressed genes, hub genes

## Abstract

**Background:** Primary biliary cholangitis (PBC) is a chronic autoimmune liver disease that eventually progresses to cirrhosis and hepatocellular carcinoma (HCC) in the absence of proper treatment. However, Gene expression and molecular mechanisms involved in the pathogenesis of PBC have not been completely elucidated.

**Methods:** Microarray expression profiling dataset GSE61260 was downloaded from the Gene Expression Omnibus (GEO) database. Data were normalized to screen differentially expressed genes (DEGs) using the limma package in R. Moreover, Gene Ontology (GO) and Kyoto Encyclopedia of Genes and Genomes pathway (KEGG) enrichment analyses were performed. A protein–protein interaction (PPI) network was constructed to identify hub genes and an integrative regulatory network of transcriptional factor–DEG–microRNA was established. Gene Set Enrichment Analysis (GSEA) was used to analyze differences in biological states for groups with different expressions of aldo-keto reductase family 1 member B10 (AKR1B10). Immunohistochemistry (IHC) analysis was performed to validate the expression of hepatic AKR1B10 in patients with PBC. The association of hepatic AKR1B10 levels with clinical parameters was evaluated using one-way analysis of variance (ANOVA) and Pearson’s correlation analysis.

**Results:** This study identified 22 upregulated and 12 downregulated DEGs between patients with PBC and healthy controls. GO and KEGG analysis revealed that DEGs were mainly enriched in immune reactions. AKR1B10 was identified as a key gene and was further analyzed by screening out hub genes from the PPI network. GSEA analysis indicated that high expression of AKR1B10 might promote PBC to develop into HCC. Immunohistochemistry results verified the increased expression of hepatic AKR1B10 in patients with PBC and demonstrated its positive correlation with the severity of PBC.

**Conclusion:** AKR1B10 was identified as a hub gene in PBC by integrated bioinformatics analysis and clinical validation. The increase of AKR1B10 expression in patients with PBC was associated with disease severity and might promote the progression of PBC to HCC.

## 1 Introduction

Primary biliary cholangitis (previously named primary biliary cirrhosis, PBC) is a chronic cholestatic liver disease characterized by a female predominance, production of specific anti-mitochondrial antibodies (AMAs), and immunological destruction of small- and medium-sized bile ducts (Kaplan and Gershwin, 2005; Gulamhusein and Hirschfield, 2020). Untreated PBC is progressive and may lead to fibrosis and cirrhosis and even liver failure and hepatocellular carcinoma (HCC) (Tanaka, 2021). Globally, the prevalence and incidence of PBC vary greatly and have been increasing. Approximately 1 in 1000 women over the age of 40 years live with PBC (Jepsen et al., 2015). Only ursodeoxycholic acid (UDCA) and obeticholic acid (OCA) have been approved by the United States Food and Drug Administration for treating PBC. However, ∼40% of patients with PBC respond inadequately to UDCA (Carey et al., 2015), and OCA was reported to increase the risk of liver failure (Chascsa and Lindor, 2020; Eaton et al., 2020). Therefore, a more in-depth understanding of the molecular mechanism involved in the development and progression of PBC is urgently needed to facilitate the development of therapeutic targets.

The pathogenesis of PBC is complex, involving multiple factors, such as environmental factors and epigenetic regulation. The loss of tolerance to biliary epithelial cells, driven by the dysregulation of the innate and adaptive immune pathways, is the initiating event of PBC (Gulamhusein and Hirschfield, 2020). However, because obtaining clinical samples of PBC is challenging and suitable animal models of PBC are lacking, the pathophysiology of PBC is unclear. High-throughput sequencing technologies and bioinformatic analysis have been widely used to study gene networks and identify key genes involved in various diseases; however, their application in PBC is limited.

In this study, microarray gene expression profiles (GSE61260)—from the liver tissues of patients with PBC—were obtained from the Gene Expression Omnibus (GEO) database. The limma package in R was used to screen out differentially expressed genes (DEGs) between patients with PBC and healthy controls (HCs). Subsequently, Gene Ontology (GO) and Kyoto Encyclopedia of Genes and Genomes (KEGG) enrichment analysis, and protein–protein interaction (PPI) network analysis were performed to elucidate the molecular function and interaction of the DEGs. In addition, integrative regulatory networks of transcription factors–DEGs–microRNAs were constructed to reveal the regulatory mechanisms of key genes in PBC. Finally, a hub gene aldo-keto reductase family 1 member B10 (AKR1B10), which has not been reported to be related to PBC, was identified. The expression of AKR1B10 and its correlation with the severity of PBC in clinical samples were analyzed. This study identified a potential gene involved in PBC, enhanced the understanding of the molecular mechanisms involved in the progression of the disease, and may provide insights for the development of therapeutic interventions for patients with PBC.

## 2 Materials and methods

### 2.1 Data acquisition and processing

The dataset (GSE61260), containing 11 liver tissue samples of patients with PBC and 38 liver tissue samples of HCs, was downloaded from the GEO database (http://www.ncbi.nlm.nih.gov/geo/). According to the annotation information in the platform, all IDs were labeled with the corresponding gene symbols, and the mean value method was used to remove duplicate gene names.

### 2.2 Identification of DEGs

DEGs were screened out in all datasets using the limma package. Adjusted *p*-value (adj. *p*-value) < .05 and | log2 fold change (FC) | ≥ 1 were considered threshold values of significant DEGs.

### 2.3 Functional enrichment analysis

The functional enrichment analysis of DEGs was performed using clusterProfiler in R. According to the expression levels of key genes, PBC was divided into high- and low-expression groups. Then, GSEA V4.2.3 was used to analyze the differences in biological states between these two groups. Molecular Signatures Database v7.5.1 provided the background gene set data required for this study. In detail, the KEGG subset of C2 and GO subset of C5 were used to perform functional enrichment analysis. FDR <25% and nominal *p* < 0.05 were considered significantly enriched.

### 2.4 Establishment of PPI networks and identification of hub genes

PPI networks of DEGs were evaluated using STRING. Cytoscape V3.9.1 was used to visualize the PPI network of key DEGs. MCODE in Cytoscape was used to screen out the hub cluster.

### 2.5 Transcription factor–DEG–microRNA network construction

MiRTarBase v8.0 and ENCODE were used to obtain TF–DEG and miRNA-DEG interaction data. DEGs showing reciprocal changes in transcription factor (TF) and microRNA (miRNA) were selected, and the miRNAs and TFs that regulate them were extracted. Finally, the integrated TF–DEG–miRNA network was obtained and visualized in Cytoscape.

### 2.6 Human liver samples

A total of 59 paraffin-embedded liver sections, including samples from 18 HCs to 41 patients with PBC, were obtained from Xiangya Hospital (Changsha, Hunan, China) between February 2017 and September 2022. Patients with PBC were diagnosed by experienced physicians based on levels of serum alkaline phosphatase (ALP), status of serum AMAs, and histological evaluation of biopsied liver tissue. The histological sections were evaluated by experienced hepatopathologists in a blinded fashion and the stages of PBC, degree of fibrosis, and bile duct loss in each sample were scored using the Ludwig (Ludwig et al., 1978) and Nakanuma systems (Nakanuma et al., 2010). The control liver sections were obtained from normal liver tissues without unusual histological features, such as hepatic hemangioma, and nontumoral parts of livers complicated with a liver tumor. The detailed clinical characteristics of the enrolled patients are listed in Supplementary Table S1.

This study was approved by the Ethics Review Board of Xiangya Hospital Central South University (No. 20221025). Written informed consent was obtained from all participants.

### 2.7 Immunohistochemistry analysis

Immunohistochemistry (IHC) analysis was performed as described (Wang et al., 2022). In brief, the paraffin-embedded sections were deparaffinized and hydrated. Antigen retrieval was achieved using Tris-EDTA buffer at pH 9.0. After endogenous peroxidase activity was blocked, the sections were incubated with an anti-AKR1B10 antibody (cat#ab192865, Abcam, Cambridge, United Kingdom) at a dilution of 1:400 at 4°C overnight. Primary rabbit IgG antibody (cat#ab172730, Abcam, Cambridge, United Kingdom) was used as the negative control. Staining was visualized by using 3,3′-diaminobenzidine tetrahydrochloride (cat#SP-9000, ZSBio, Beijing, China) and hematoxylin counterstain. Representative images were captured under a light microscope (Leica, DMIL LED, Wetzlar, Germany; magnification, ×200). The intensity of IHC staining was designated 0 for no staining, 1 for weak staining, 2 for moderate staining, and 3 for strong staining. AKR1B10 immunostaining was based on the positive cytoplasmic staining and was quantitatively assessed as the average percentage of AKR1B10-positive areas in three independent fields. The extent of stained areas was determined as the percentage of stained areas, ranging from 0% to 100%. The IHC score was obtained by multiplying intensity scores with proportion scores, as described (Qu et al., 2018).

### 2.8 Statistical analysis

Data are represented as mean ± standard deviation (SD). All data were statistically analyzed using GraphPad Prism V9.1.1. Significance of differences between two groups was determined by two-tailed independent-sample Student’s t-test. Differences between more than two groups were evaluated using one-way analysis of variance (ANOVA). Pearson’s correlation coefficient was used to evaluate the correlation between the two factors. Differences were considered significant at bilateral *p* < .05.

## 3 Results

### 3.1 Data normalization

**FIGURE 1:**
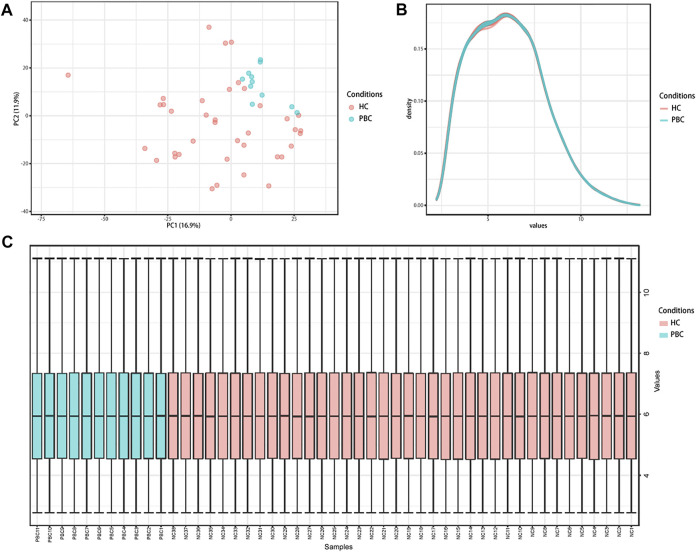
Distribution features of the expression situation of samples after normalization. **(A)** PCA shows the distribution of expression values of all samples. **(B)** The density curve of all samples. **(C)** Box plot comparing expression values of all samples. PCA, principal component analysis; PBC, Primary biliary cholangitis; HC, healthy control.

Microarray dataset GSE61260 was obtained from the GEO database. A total of 49 liver tissue samples (11 PBC and 38 HC) were included in this dataset. Data normalization and cross-comparability were applied to eliminate technical and systematic variability. Principal component analysis (PCA) was performed to verify biological variability between each sample. The PCA plot showed that PBC and HC samples were grouped separately, indicating distinct gene expression profiles (Figure 1A). The density plot suggested that the gene expression values of samples from different groups followed nearly the same curve and could be used for subsequent analysis (Figure 1B). The box plot showed the gene expression range of each sample, with the black lines in the boxes almost on the same straight line (Figure 1C). Raw data were normalized robustly, ensuring data reliability for downstream analysis.

### 3.2 Identification of DEGs

**FIGURE 2:**
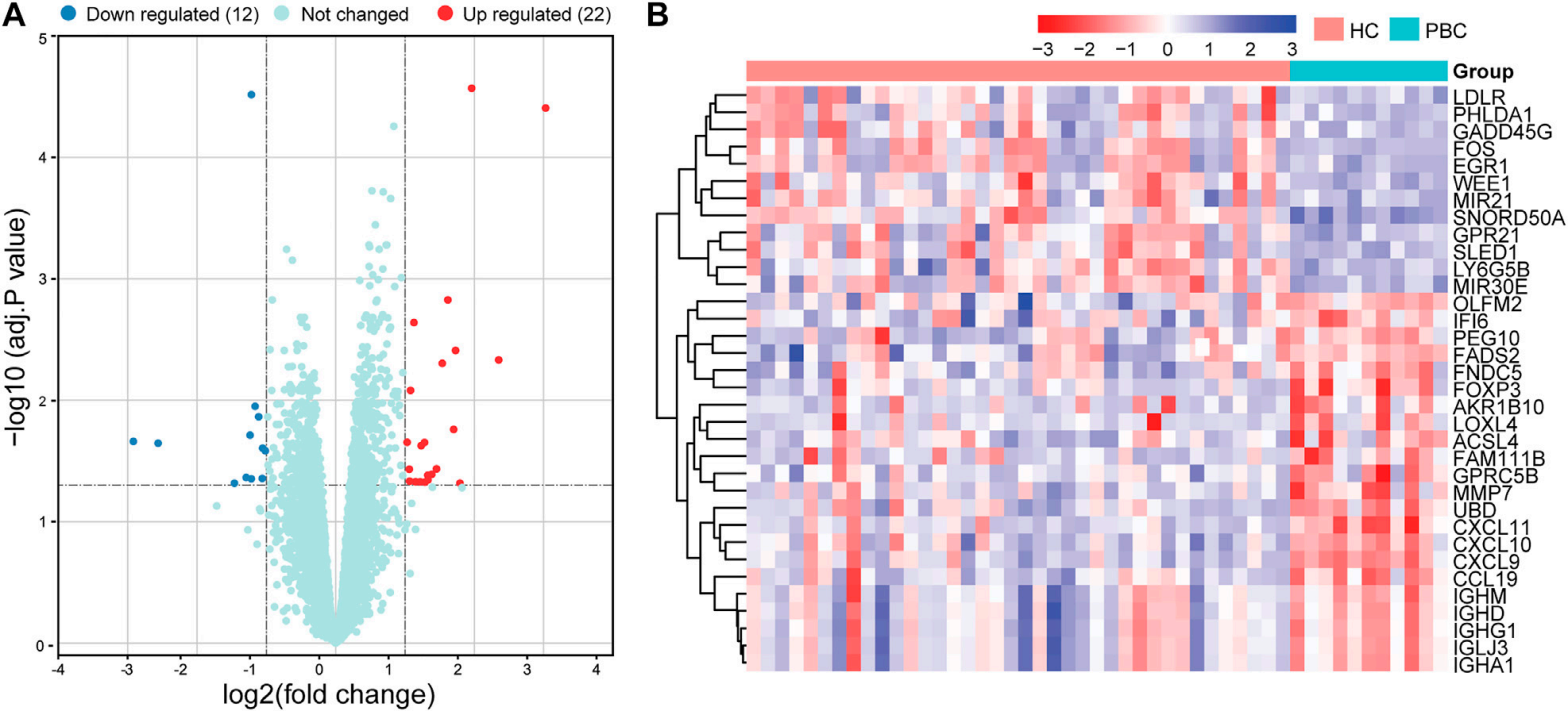
Volcano plot and heatmap of DEGs in the datasets. **(A)** Volcano map of all DEGs in GSE61260 analyzed using the limma package in R. Adjusted *p*-value <.05 and | log2 FC | ≥ 1 were considered threshold values of significant DEGs. **(B)** Heatmap of DEGs, including 22 up- and 12 downregulated genes. DEGs, differentially expressed genes; PBC, Primary biliary cholangitis; HC, healthy control.

Based on the limma package in R language (|logFC| ≥1, adj. *p*-value < .05), 34 DEGs were identified, including 22 up- and 12 downregulated DEGs (Supplementary Table S2). The volcano map of DEGs is presented in Figure 2A and the heatmap in Figure 2B. Significant differences in gene expression were observed between the two groups.

### 3.3 Functional enrichment analysis of DEGs

**FIGURE 3:**
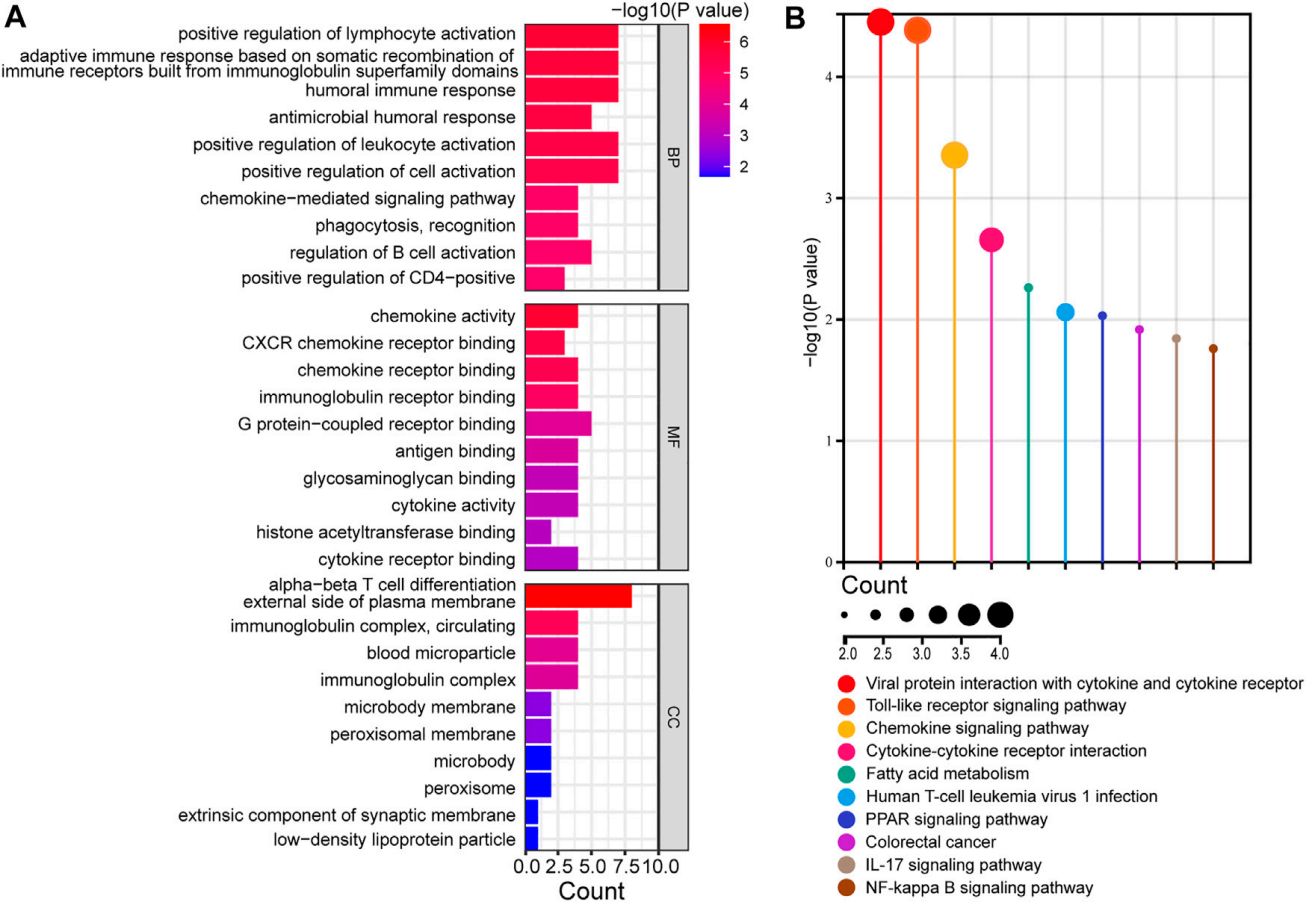
Functional enrichment analysis of DEGs. **(A)** Analysis of GO terms, including BPs, MFs, and CCs, of DEGs. **(B)** KEGG analysis of DEGs. GO, gene ontology; DEGs, differentially expressed genes; BP, biological process; MF, molecular function; CC, cellular components; KEGG, Kyoto encyclopedia of genes and genomes.

To investigate the biological functions of the identified DEGs, GO enrichment analysis was performed using clusterProfiler (Figure 3A and Supplementary Table S3). The DEGs were significantly enriched in biological processes (BPs), including humoral and cellular immune responses, such as the activation of lymphocytes, leukocytes, and B cells. In molecular functions (MFs), the DEGs were mainly involved in the activity of chemokines and cytokines, as well as receptor binding of chemokines, immunoglobulins, G protein-coupled receptors, and cytokines. Cellular components (CCs) showed enrichment in the external side of the plasma membrane, immunoglobulin complex, and circulating and blood microparticles. KEGG pathway enrichment analysis was performed to identify crucial pathways of DEGs (Figure 3B and Supplementary Table S4). The DEGs were predominantly enriched in immune-related signaling pathways, such as cytokines and cytokine receptors, toll-like receptors, and chemokine signaling pathways. Overall, the function of DEGs was markedly associated with immune reaction. These results indicated that immune and inflammatory processes characterize the development of PBC, which is consistent with other findings (Lleo et al., 2020).

### 3.4 PPI network construction of DEGs

**FIGURE 4:**
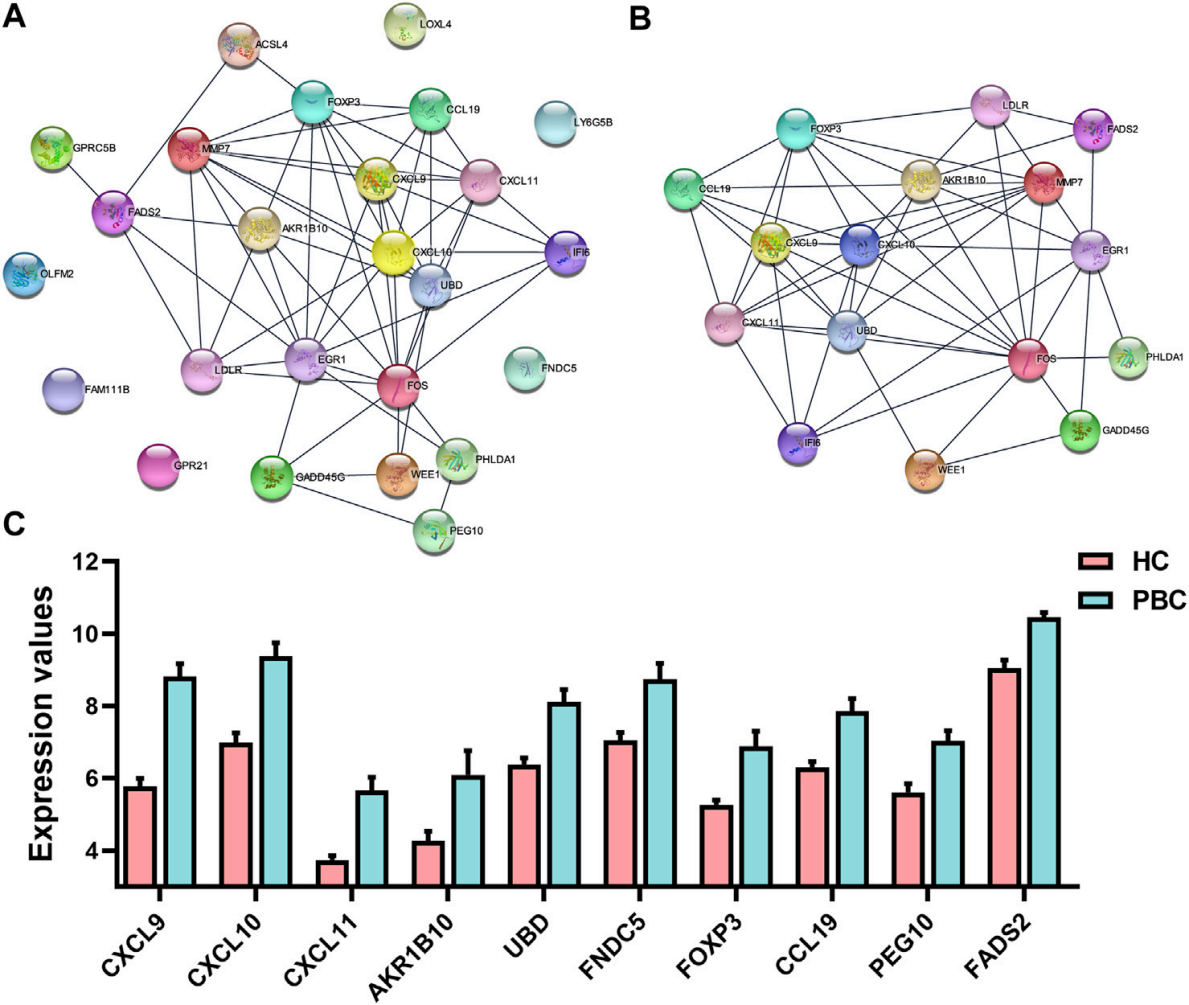
The PPI network and the most significant module of DEGs. **(A)** The PPI network of DEGs was constructed using STRING. **(B)** The most significant module was obtained from the PPI network using MCODE in Cytoscape. **(C)** Expression changes of top 10 DEGs between PBC and HC samples based on the microarray dataset GSE61260. PPI, protein–protein interaction; DEGs, differentially expressed genes; PBC, Primary biliary cholangitis; HC, healthy control.

The STRING database was used to construct the PPI network, which can illustrate the relationship of the interaction between DEG-coded proteins. The PPI network contained 25 nodes and 59 edges (Figure 4A). Most DEG-coded proteins were highly connected with others. Moreover, the most significant module, which included 16 genes, was screened out from the PPI network based on module analysis using MCODE (Figure 4B). The DEGs in the module included AKR1B10, CXCL9, CXCL10, CXCL11, UBD, FOXP3, MMP7, CCL9, EGR1, LDLR, FADS2, FOS, IFI6, WEE1, PHLDA1, and GADD45G. Moreover, changes in the expression of the top 10 genes differed strikingly between PBC and HC samples according to the GEO database (Figure 4C). The top 3 DEGs, including CXCL9, CXCL10, and CXCL11, have been widely studied in the pathogenesis of PBC (Chuang et al., 2005; Tacke et al., 2011; Manousou et al., 2013). However, the fourth DEG AKR1B10 was the focus of this study. The findings revealed that not only was AKR1B10 a notably upregulated DEG in PBC, but also one of the hub genes in the PPI network of the DEGs.

### 3.5 Construction of the TF–DEG–miRNA network

**FIGURE 5:**
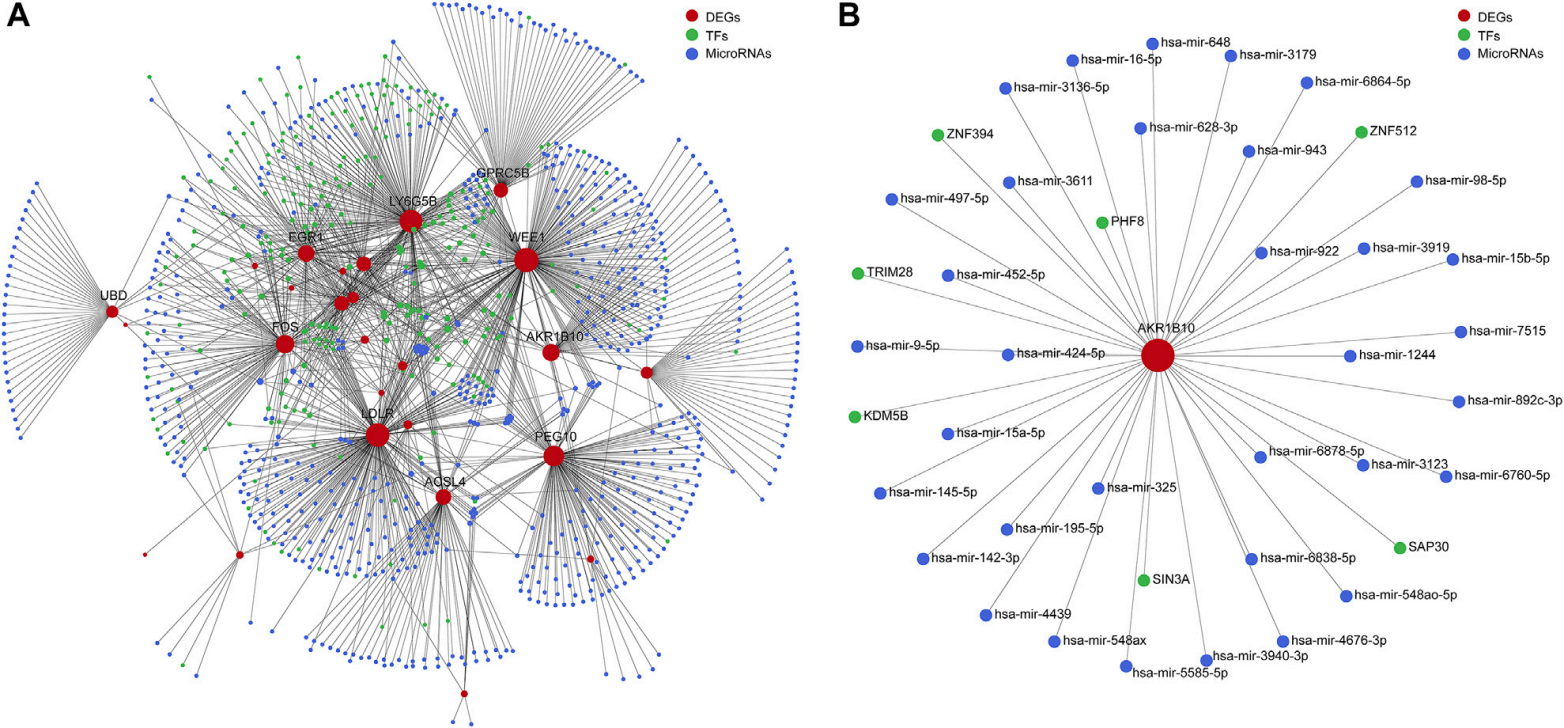
Integrative regulatory network analysis. **(A)** The TF–DEG–miRNA integrative regulatory network. **(B)** Integrative regulatory network analysis of AKR1B10. TF, transcription factor; DEGs, differentially expressed genes; miRNA, microRNA; AKR1B10, aldo-keto reductase family 1 member B10.

To investigate the functional roles of DEGs, the potential regulatory relationship between DEGs and TFs, DEGs, and miRNAs were explored. The miRNA–DEG pairs were identified through the network analysis of DEGs using the miRTarBase database. The potential regulatory relationships between DEGs and TFs were screened based on TF-binding site data and genetic coordinate position information provided on ENCODE. The merged TF–DEG–miRNA interaction network included 569 nodes and 1370 edges (Figure 5A). Notably, AKR1B10 was predicted to regulated by 7 TFs (KDM5B, TRIM28, PHF8, ZNF394, ZNF512, SAP30, and SIN3A) and 34 interacting miRNAs, such as has-mir-98-5p, has-mir-452-5p, and has-mir-497-5p (Figure 5B).

### 3.6 Functional enrichment analysis of PBC samples with differential AKR1B10 expression

**FIGURE 6:**
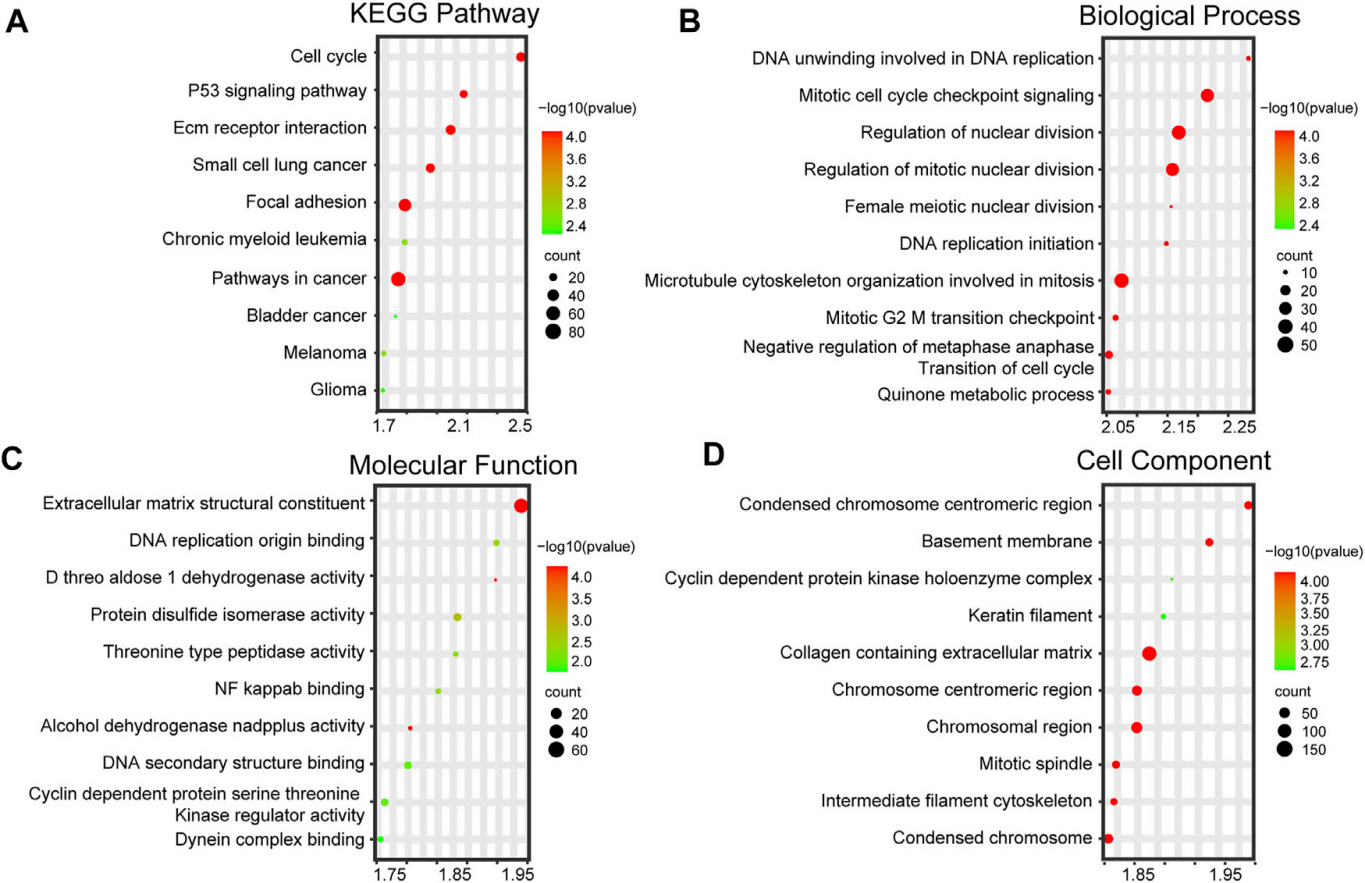
Functional enrichment analysis of PBC with differential AKR1B10 levels. High and low AKR1B10 groups are based on the median of AKR1B10 expression levels. **(A)** Top 10 KEGG pathways of the AKR1B10 high-expression group. Top 10 enriched GO terms, including BPs **(B)**, MFs **(C),** and CCs **(D)**, of the AKR1B10 high-expression group. AKR1B10, aldo-keto reductase family 1 member B10; PBC, Primary biliary cholangitis; KEGG, Kyoto encyclopedia of genes and genomes; GO, Gene Ontology; BP, biological process; MF, molecular function; CC, cellular component.

To further study the biological functions of AKR1B10 in PBC, 11 samples of PBC in GSE61260 were divided into the AKR1B10 low- (6 samples) and AKR1B10 high-expression (5 samples) groups, according to the median expression levels of AKR1B10. GSEA was applied to the functional enrichment analysis of the two groups. Importantly, KEGG enrichment analysis suggested that all the enriched signaling pathways of the AKR1B10 high-expression group were associated with cancer, including the cell cycle, P53 signaling pathway, and pathways in cancer (Figure 6A and Supplementary Table S5). GO enrichment analysis (including BP, MF, and CC) collectively showed that the AKR1B10 high-expression group was largely involved in cell proliferation, including DNA replication, nuclear division, and cell cycle transition (Figures 6B–D and Supplementary Table S6). These results indicate that high expression of AKR1B10 might improve the progression from PBC to HCC by regulating cancer-related pathways.

### 3.7 Validating the expression of AKR1B10 in clinical samples

**FIGURE 7:**
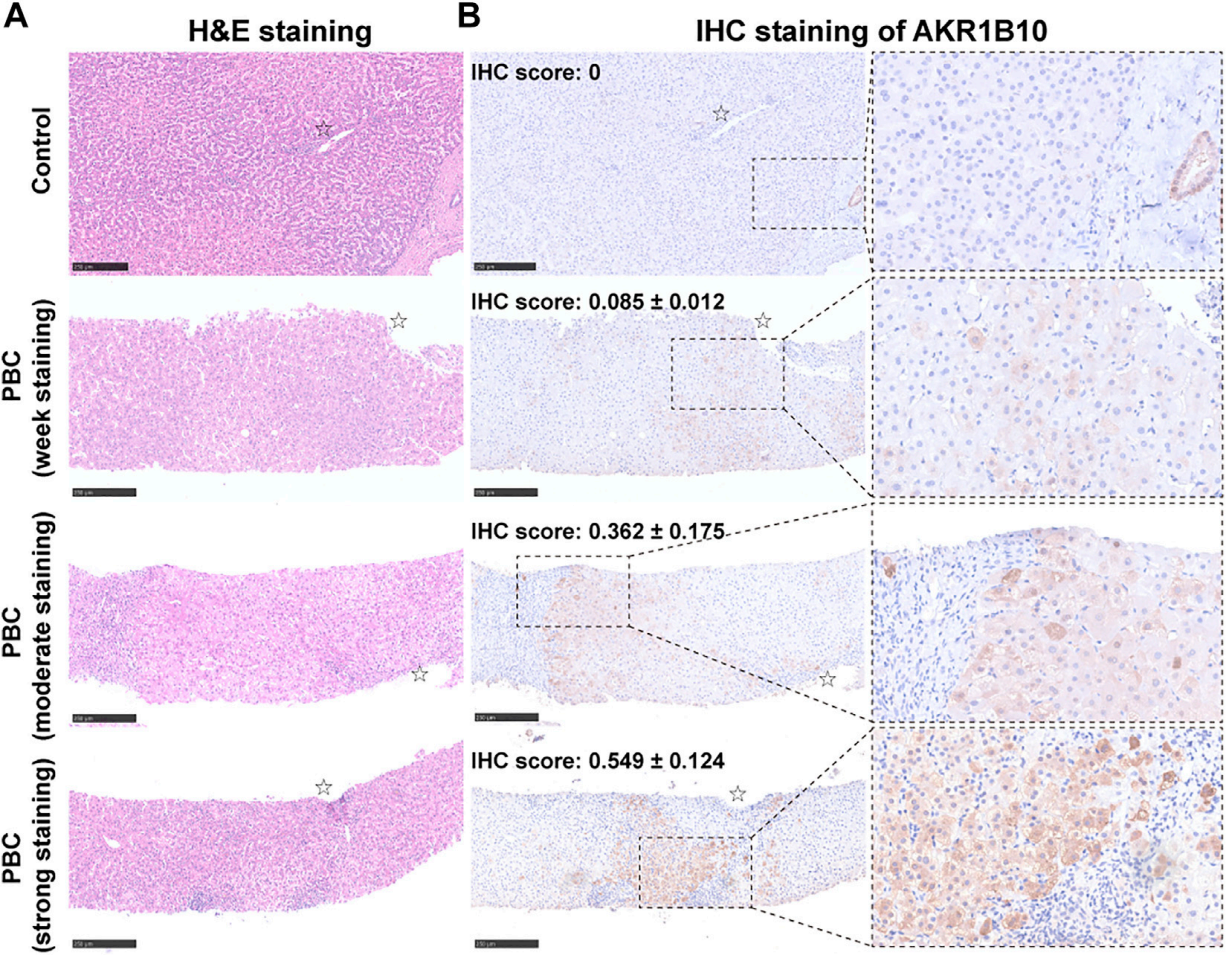
Histological and immunohistochemistry analyses of serial liver sections from patients with PBC and control patients. Representative images of hematoxylin and eosin (H&E) staining **(A)** and immunohistochemical staining of AKR1B10 with different staining intensities **(B)** in serial liver sections from healthy controls and patients with PBC. Scale bars: 250 μm. AKR1B10, aldo-keto reductase family 1 member B10; PBC, Primary biliary cholangitis.

**FIGURE 8:**
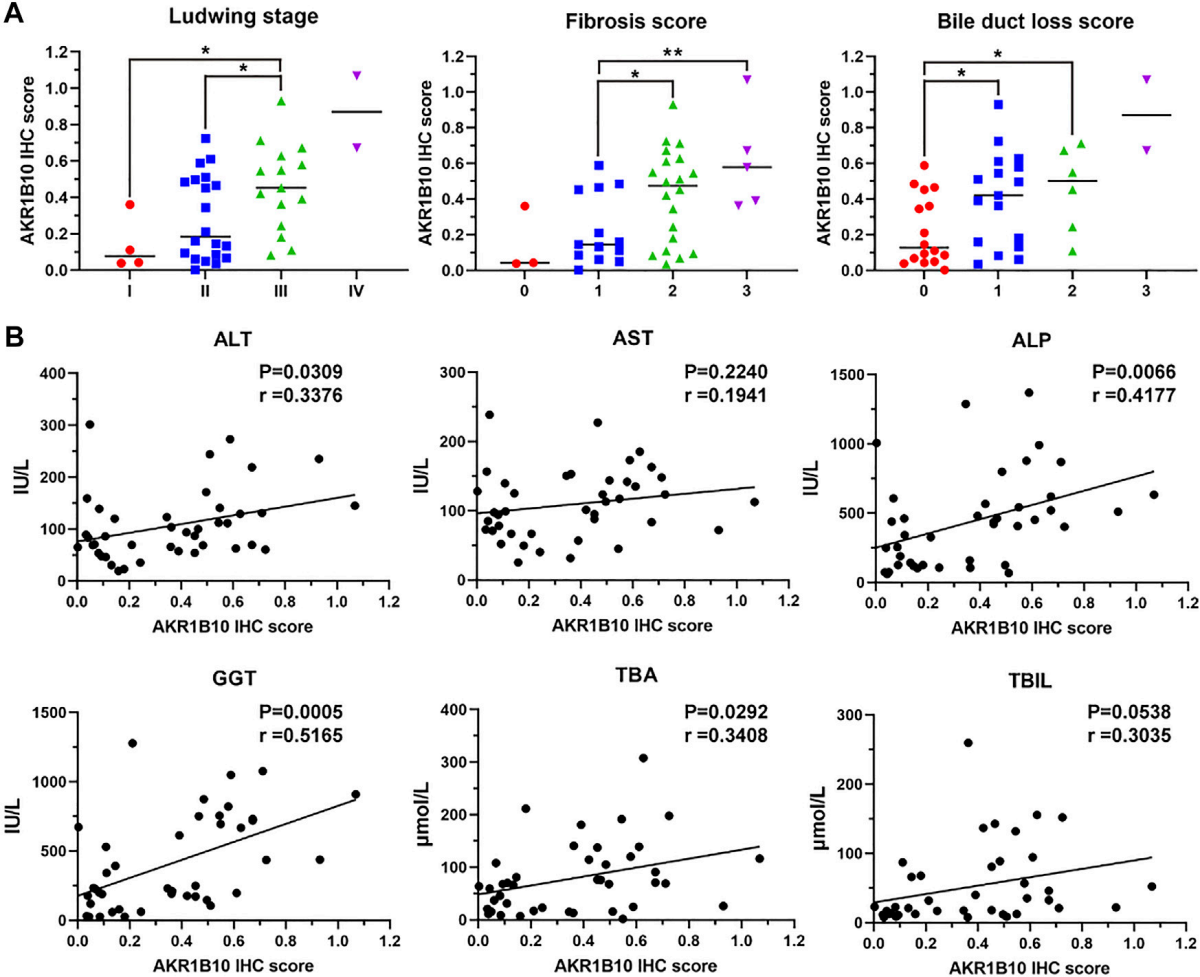
Relationship between hepatic AKR1B10 expression and clinicopathological characteristics in patients with PBC. **(A)** The relationship between hepatic AKR1B10 expression and the Ludwig stage, degree of fibrosis, and bile duct loss scores in patients with PBC. Plotted: Mean ± S.D.; Statistics: one-way analysis of variance (ANOVA); *p < 0.05. **(B)** The correlations between hepatic AKR1B10 expression and clinicopathological features in patients with PBC. Statistics: Pearson’s correlation analysis. AKR1B10, aldo-keto reductase family 1 member B10; PBC, Primary biliary cholangitis; ALT, alanine aminotransferase; AST, aspartate aminotransferase; ALP, alkaline phosphatase; GGT, gamma-glutamyl transferase; TBA, total bile salts; TBIL, total bilirubin.

To further investigate AKR1B10 expression and localization in patients with PBC, liver tissue samples of 18 HCs and 41 patients with PBC were collected. For patients with PBC, the age ranged from 24 to 70 years (mean = 50 years), with a strong female predominance (82.93% female vs. 17.07% male) (Table 1 and Supplementary Table S1). IHC analysis revealed that AKR1B10 was almost undetectable in hepatocytes; however, it was mainly observed in the bile duct of HCs (Figures 7A, B), which is consistent with other reports (Murata et al., 2016). By contrast, AKR1B10 was predominantly expressed in the hepatocytes around the periportal area with different staining intensities and proportions in patients with PBC (Figures 7A, B). Notably, higher AKR1B10 expression scores were markedly associated with the severity of PBC, including higher Ludwig stages, fibrosis scores, and bile duct loss scores (Figure 8A). Regression analysis showed a significant correlation between AKR1B10 immunoreactivity and serum ALT (*r* = .3376, *p* = .0309), ALP (*r* = .4177, *p* = .0066), GGT (*r* = .5165, *p* = .0005), and TBA (*r* = .3408, *p* = .0292) levels (Figure 8B). These data indicated that the expression of hepatic AKR1B10 is substantially increased in patients with PBC and is significantly associated with the severity of PBC.

**TABLE 1:** Clinical features of patients.

Clinical features	Healthy controls	Patients with PBC
Total samples (Male/Female)	18 (9/9)	41 (7/34)
Age (years)	52 ± 11	50 ± 9
ALT (IU/L)	27.0 ± 19.6	106.5 ± 67.6*
AST (IU/L)	24.7 ± 8.6	109.1 ± 49.6*
ALP (IU/L)	78.2 ± 18.6	436.9 ± 334.2†
GGT (IU/L)	43.8 ± 30.3	412.0 ± 341.3†
TBA (μmol/L)	7.8 ± 8.2	79.2 ± 67.7*
TBIL (μmol/L)	12.8 ± 5.7	50.9 ± 55.5†
DBIL (μmol/L)	5.1 ± 2.5	31.6 ± 35.6†

Values are means ± SD., Statistics: two-tailed independent-sample Student’s t-test; *p < .001, †*p* < .01 *versus* controls.

PBC, primary biliary cholangitis; ALT, alanine aminotransferase; AST, aspartate aminotransferase; ALP, alkaline phosphatase; GGT, gamma-glutamyl transferase; TBA, total bile salts; TBIL, total bilirubin; DBIL, direct bilirubin.

## 4 Discussion

Although mortality due to PBC has reduced, its prevalence has increased (Lu et al., 2018). The management of PBC is limited. Current therapeutic methods cannot help in achieving a “cure” for patients with PBC, and the patients must take medicines life-long. For patients that show incomplete response to UDCA and second-line treatment, such as OCA, liver transplantation is the only treatment option (Shah and Kowdley, 2020). These unresolved needs underscore the urgency to improve our understanding of the pathogenesis and molecular mechanisms of disease progression.

This study highlights the following major findings: 1) AKR1B10 was identified as a key gene in the progression of PBC using bioinformatics analysis; 2) high expression of hepatic AKR1B10 might promote the progression of PBC to HCC; 3) increase in hepatic AKR1B10 expression was associated with PBC severity; 4) levels of hepatic AKR1B10 were positively correlated with the circulatory parameters associated with hepatic function in patients with PBC.

AKR1B10 is a member of the aldo-keto reductase superfamily, a multifunctional nicotinamide adenine dinucleotide phosphate (NADPH)-dependent oxidoreductase that metabolizes endogenous carbonyl compounds and xenobiotics (Barski et al., 2008). This function is critical for protecting host cells from DNA damage induced by electrophilic carbonyl compounds (Zu et al., 2017). Growing evidence supports that AKR1B10 is involved in the occurrence and progression of many cancers, such as HCC (Ye et al., 2019), breast cancer (Qu et al., 2021), non-small cell lung cancer (Fukumoto et al., 2005), and pancreatic cancer (Chung et al., 2012). Because of its role as an effective retinal reductase, AKR1B10 is considered to have an inhibitory effect on retinoic acid signaling, which results in cellular proliferation (Gallego et al., 2007). Therefore, the up-regulation of AKR1B10 is believed to play a key role in promoting the tumor phenotype of cancer cells (Ruiz et al., 2012). In addition, AKR1B10 may be upregulated by the activation of some signal transduction pathways, such as MAPK (Nishinaka et al., 2015), Nrf2 (Nishinaka et al., 2017), and AP-1 (Cheng et al., 2018) pathways, and promote the proliferation and migration of cancer cells. Moreover, given its role in detoxification, AKR1B10 was shown to contribute to the chemoresistance acquisition against some anti-cancer drugs, such as daunorubicin, and idarubicin (Zhong et al., 2011) (Morikawa et al., 2015), and cisplatin (Matsunaga et al., 2016).

AKR1B10 was shown to be upregulated not only in cancers but also in some preneoplastic conditions, such as Barrett’s esophagus (Breton et al., 2008) and squamous metaplasia (Li et al., 2008). Importantly, AKR1B10 was reportedly upregulated in some chronic liver diseases that are thought to be preneoplastic diseases of HCC. Two similar studies have demonstrated that patients with chronic hepatitis B (Li et al., 2008) and chronic hepatitis C (Murata et al., 2016) expressed high levels of AKR1B10 and its expression acted as an independent risk factor for HCC development. AKR1B10 expression was reported to be significantly increased in nonalcoholic steatohepatitis, where it promotes the progression of nonalcoholic steatohepatitis to HCC through several mechanisms, such as by contributing to lipogenesis and facilitating the detoxification of reactive carbonyl species derived from lipid peroxidation (Bitter et al., 2015; Endo et al., 2021). Over the past decade, AKR1B10 has emerged as a promising biomarker for HCC. Although the molecular mechanisms underlying hepatocarcinogenesis remain poorly clarified, AKR1B10 may play a pivotal role in HCC through several mechanisms, including lipogenesis, oxidative stress, detoxification of cytotoxic reactive carbonyls, and regulation of sphingosine-1 phosphate and retinoic acid (DiStefano and Davis, 2019). Considering that higher expression of AKR1B10 is correlated with increased survival rate and lower metastatic incidence, AKR1B10 acts as a useful biomarker for the prognosis of HCC (Liu et al., 2015) (Jin et al., 2016).

In addition to improvements in long-term outcomes for patients with PBC, the development of HCC is not a rare event. A previous meta-analysis, with a larger sample size and stronger evidence, demonstrated an increased risk of HCC in PBC patients, which was more than 18.8-fold higher than that of the general population (Liang et al., 2012). Male sex, advanced histological stage, and non-response to UDCA are defined as high-risk factors that contribute to the development of HCC from PBC (Tanaka, 2021). The findings of this study demonstrate for the first time that AKR1B10 is a hub gene that is highly expressed in patients with PBC. GSEA analysis in the AKR1B10 high- and low-expression groups revealed that AKR1B10 may promote the progression of PBC to HCC. However, PBC predominantly occurs in women, which is contrary to HCC. The incidence rates of HCC are up to 2-3 fold higher in men compared to women (Yang et al., 2020). One of the reasons for the gender disparity is that hepatitis B virus (HBV) is the major etiology for HCC. As the sixth most commonly diagnosed cancer and the fourth leading cause of cancer death worldwide, the etiologies for HCC are diverse, in which HBV plays a dominant role (the percentage of HCC by HBV is 41%) (Liu et al., 2022). Chronic HBV infection is more common in men compared to in women (10.7% vs. 4.4%) among those who receive vaccination at birth and are followed up for over 18 years (Su et al., 2007). Furthermore, the progression of HBV occurs faster in men than in women, which also leads to HBV-related HCC being much more common in men compared to women (with a ratio of 5–7:1) (Lee et al., 1999; Zheng et al., 2017). The above reports suggested that men have a greater risk of developing HCC than women worldwide. Another reason for the gender disparity is that PBC is a relatively rare disease, so it is difficult for PBC-derived HCC to affect the gender distribution of HCC. Moreover, high levels of AKR1B10 protein in PBC livers was verified in the present study, and the increase of ARR1B10 was found to be associated with the severity of PBC. Thus, the expression of hepatic AKR1B10 is closely related to the development of PBC and affects the prognosis of patients with PBC. These findings may provide a new mechanism for the pathogenesis and progression of PBC and shed light on strategies for therapeutic intervention for patients with PBC.

Multiple studies have reported the regulatory mechanism of AKR1B10 in many diseases especially tumors. The present results revealed the regulatory network of AKR1B10 preliminarily *via* constructing the TF–AKR1B10–miRNA network. Interestingly, many TFs that potentially regulate the expression of AKR1B10 according to our analysis, such as KDM5B (Wang et al., 2016; Gong et al., 2018), TRIM28 (Zhang et al., 2021; Han et al., 2022; Zhang et al., 2022), and PHF8(Zhou et al., 2018), had been previously identified to promote the development of HCC. Considering AKR1B10 is suggested to facilitate the progression of PBC to HCC, we speculate that AKR1B10 may be regulated by these TFs during this process. Furthermore, miRNA plays another significant role in regulating the expression of targeted genes. A previous study suggested that miR-98, a miRNA also predicted in our analysis, could regulate AKR1B10–ERK signaling (Cong et al., 2019). Importantly, ERK signaling plays a pivotal role in cholestatic liver diseases including PBC (Jones et al., 2015; Chen L. et al., 2021; Chen X. et al., 2021; Wang et al., 2022). Therefore, miR-98–AKR1B10–ERK signaling is probably also involved in PBC. However, further research needs to be performed to elucidate the contribution of these potential genes to AKR1B10 in PBC. In addition to the genes discussed above, it is also worth exploring whether other regulators may interact with AKR1B10 in PBC.

This study has some limitations. First, the microarray data were obtained from public datasets and not generated by the authors. Second, the levels of AKR1B10 in the blood samples of patients with PBC have not been confirmed. This will be an area for further research because it may provide rapid, accessible strategies for the diagnosis and prognosis of PBC. Finally, the function and molecular mechanisms involving AKR1B10 in the development and progression of PBC remain unclear. In the future, in-depth analysis using *in vivo* and *in vitro* experiments is warranted.

## 5 Conclusion

In summary, this study identifies AKR1B10 as a key gene that correlates with the progression of PBC, using bioinformatics analysis combined with clinical validation. The findings of this study provide an insight into gene expression and molecular mechanisms involved in the development and progression of PBC. In the future, experiments at the cellular and molecular levels are required to verify the biological function of AKR1B10 in PBC.

## Data Availability

The original contributions presented in the study are included in the article/Supplementary Material, further inquiries can be directed to the corresponding authors.
