# Supplementary material for: Identification of AKR1B10 as a key gene in primary biliary cholangitis by integrated bioinformatics analysis and experimental validation
Source: Front Mol Biosci. 2023 Feb 9;10:1124956. doi: 10.3389/fmolb.2023.1124956 (PMC9947156; doi:10.3389/fmolb.2023.1124956)
Supplement: Supplementary file 1 [file Table1.DOCX]

Supplementary Material

Identification of AKR1B10 as a Key Gene in Primary Biliary Cholangitis by Integrated Bioinformatics Analysis and Experimental Validation

Huiwen Wang^1†^, Jian Zhang^1†^, Jingqin Liu^1^, Yongfang Jiang^2^, Lei Fu^1^*, Shifang Peng^1^*

^1^Department of Infectious Diseases, Xiangya Hospital, Central South University, Changsha, China

^2^Department of Infectious Diseases, Second Xiangya Hospital, Central South University, Changsha, China

*** Correspondence:**

Shifang Peng

[sfp1988@csu.edu.cn](mailto:sfp1988@csu.edu.cn)

Lei Fu

fulei92@126.com

**^†^**These authors have contributed equally to this work

**Supplementary Table 1.** **The detailed clinical characteristics of patients for Immunohistochemistry analysis analyses**

| **ID** | **Gender, Age** | **AMA-M2** | **Ludwing stage** | **Fibrosis score** | **BD loss score** | **ALT**  **(IU/L)** | **AST**  **(IU/L)** | **ALP**  **(IU/L)** | **GGT**  **(IU/L)** | **TBIL**  **(μmol/L)** | **DBIL**  **(μmol/L)** | **TBA**  **(μmol/L)** | **AKR1B10**  **IHC Score** |
| --- | --- | --- | --- | --- | --- | --- | --- | --- | --- | --- | --- | --- | --- |
| PBC (1) | F, 43y | + | Ⅳ | 3 | 3 | 145.1 | 112.5 | 633.3 | 909.5 | 52.4 | 29.1 | 116.4 | 1.068 |
| PBC (2) | F, 54y | + | Ⅲ | 2 | 1 | 235 | 72.1 | 510.3 | 437.9 | 22 | 14 | 26.1 | 0.93 |
| PBC (3) | M, 50y | + | Ⅱ | 2 | 1 | 60.6 | 123.5 | 401.6 | 435.2 | 152 | 117 | 197.7 | 0.724 |
| PBC (4) | F, 52y | N.D. | Ⅲ | 2 | 2 | 131 | 148 | 869 | 1077 | 21 | 11 | 69.3 | 0.711 |
| PBC (5) | F, 46y | - | Ⅳ | 3 | 3 | 219 | 163 | 521 | 732 | 46 | 38 | 70.9 | 0.672 |
| PBC (6) | F, 63y | N.D. | Ⅲ | 2 | 2 | 69.4 | 83.7 | 621.2 | 720.7 | 32.6 | 19.2 | 90.8 | 0.672 |
| PBC (7) | F, 51y | + | Ⅲ | 2 | 1 | 129.5 | 185.4 | 991.6 | 667.8 | 155.6 | 95.9 | 307.7 | 0.627 |
| PBC (8) | F, 63y | - | Ⅱ | 2 | 1 | 62.9 | 135.2 | 451.3 | 197 | 94.7 | 54.2 | 139 | 0.61 |
| PBC (9) | F, 29y | + | Ⅱ | 1 | 0 | 273.1 | 173.1 | 1369.5 | 1048.8 | 35.3 | 18.6 | 24.7 | 0.588 |
| PBC (10) | M, 34y | - | Ⅲ | 3 | 1 | 111.4 | 141.8 | 877.8 | 822.2 | 56.8 | 35.1 | 120.3 | 0.578 |
| PBC (11) | F, 51y | + | Ⅲ | 2 | 2 | 140.9 | 117.4 | 543.6 | 694.7 | 12.6 | 4.6 | 1.8 | 0.549 |
| PBC (12) | F, 51y | + | Ⅲ | 2 | 1 | 112 | 45 | 407 | 755.9 | 132.2 | 72.5 | 191.6 | 0.544 |
| PBC (13) | F, 48y | + | Ⅱ | 2 | 1 | 244.2 | 143.8 | 67.9 | 105.7 | 8.6 | 4.5 | 15.8 | 0.51 |
| PBC (14) | M, 51y | + | Ⅱ | 2 | 1 | 171 | 113 | 124 | 145 | 12 | 4 | 68.2 | 0.496 |
| PBC (15) | F, 57y | + | Ⅱ | 1 | 0 | 68.8 | 123.6 | 799.5 | 874.3 | 88.8 | 57.6 | 105.3 | 0.484 |
| PBC (16) | F, 50y | - | Ⅱ | 1 | 0 | 100 | 227.3 | 462 | 751.8 | 142.9 | 86 | 75.7 | 0.465 |
| PBC (17) | F, 55y | + | Ⅱ | 1 | 0 | 54.3 | 95.2 | 435.1 | 171.6 | 80.8 | 59.7 | 137.4 | 0.452 |
| PBC (18) | M, 52y | - | Ⅲ | 2 | 2 | 87 | 88 | 422 | 249 | 18 | 7 | 76.4 | 0.452 |
| PBC (19) | M, 40y | N.D. | Ⅲ | 2 | 1 | 93.9 | 101.4 | 566.3 | 177.2 | 136.8 | 78.2 | 114.5 | 0.42 |
| PBC (20) | F, 49y | + | Ⅲ | 3 | 1 | 57.4 | 56.8 | 480.3 | 613.3 | 40.2 | 22.2 | 180.9 | 0.39 |
| PBC (21) | F, 61y | + | Ⅲ | 3 | 1 | 103.7 | 153.2 | 106 | 210 | 259.5 | 151.5 | 140.9 | 0.362 |
| PBC (22) | M, 53y | + | Ⅰ | 0 | 0 | 65.7 | 31.7 | 159.4 | 191.8 | 7.9 | 3.7 | 13.2 | 0.36 |
| PBC (23) | F, 56y | + | Ⅱ | 2 | 0 | 123.2 | 150.5 | 1288.3 | 230.4 | 17.5 | 10.1 | 15.2 | 0.345 |
| PBC (24) | F, 55y | + | Ⅲ | 2 | 2 | 35.4 | 40.4 | 107 | 62.2 | 17 | 7.9 | 23.3 | 0.243 |
| PBC (25) | F, 48y | + | Ⅱ | 1 | 0 | 69.4 | 66.8 | 326.6 | 1278.1 | 32 | 19.5 | 17.2 | 0.21 |
| PBC (26) | F, 70y | + | Ⅲ | 2 | 2 | 23.3 | 49.5 | 125.4 | 25.4 | 67.8 | 33.0 | 211.4 | 0.18 |
| PBC (27) | F, 67y | + | Ⅱ | 2 | 1 | 19.4 | 25.4 | 103.4 | 78.0 | 12.7 | 6.0 | 7.3 | 0.159 |
| PBC (28) | F, 55y | + | Ⅱ | 1 | 0 | 120 | 125 | 118 | 393 | 66 | 58 | 81 | 0.144 |
| PBC (29) | F, 57y | + | Ⅱ | 1 | 1 | 30.6 | 66.8 | 141.7 | 59.3 | 21 | 8.6 | 66.2 | 0.132 |
| PBC (30) | F, 43y | - | Ⅰ | 1 | 0 | 46 | 99 | 342 | 342 | 87 | 77 | 70.1 | 0.11 |
| PBC (31) | F, 47y | N.D. | Ⅲ | 2 | 1 | 86.2 | 139.6 | 461.4 | 531.0 | 18.6 | 10.7 | 31.1 | 0.108 |
| PBC (32) | F, 53y | + | Ⅱ | 2 | 0 | 48 | 52 | 189 | 189 | 11 | 7 | 68.3 | 0.093 |
| PBC (33) | M, 46y | + | Ⅱ | 1 | 0 | 139.3 | 78.4 | 125.4 | 25.4 | 8.7 | 4.3 | 8.7 | 0.085 |
| PBC (34) | F, 41y | N.D. | Ⅲ | 2 | 1 | 53.7 | 94.1 | 254.6 | 201.2 | 22.5 | 12.7 | 45.8 | 0.082 |
| PBC (35) | F, 55y | + | Ⅱ | 2 | 0 | 70.4 | 97.5 | 606.8 | 226.4 | 13.8 | 8.1 | 108.3 | 0.067 |
| PBC (36) | F, 51y | N.D. | Ⅱ | 1 | 1 | 69.1 | 71.2 | 439.0 | 234.7 | 12.6 | 7.7 | 37.3 | 0.06 |
| PBC (37) | F, 49y | N.D. | Ⅱ | 1 | 0 | 301.4 | 238.6 | 74.1 | 119.7 | 17.1 | 7.9 | 15.6 | 0.049 |
| PBC (38) | F, 46y | + | Ⅰ | 0 | 0 | 83 | 85 | 61 | 26 | 11 | 4 | 59.6 | 0.043 |
| PBC (39) | F, 24y | + | Ⅰ | 0 | 0 | 159.1 | 156.7 | 249.5 | 176.7 | 7.9 | 2.9 | 11 | 0.038 |
| PBC (40) | F, 42y | + | Ⅱ | 2 | 1 | 89.0 | 72.9 | 73.3 | 30.3 | 11.5 | 7.1 | 20.7 | 0.034 |
| PBC (41) | F, 48y | - | Ⅱ | 1 | 0 | 65 | 128 | 1007 | 673 | 23 | 19 | 63.7 | 0.002 |
| HC (1) | M ,44y | N.D. | N.D. | N.D. | N.D. | 3.6 | 11.1 | 104.5 | 65.4 | 16.6 | 5 | 4.1 | 0 |
| HC (2) | M, 65y | N.D. | N.D. | N.D. | N.D. | 15.3 | 26.4 | 42.7 | 12 | 19.2 | 6.6 | N.D. | 0 |
| HC (3) | F, 72y | N.D. | N.D. | N.D. | N.D. | 27.1 | 30 | 67.7 | 93.8 | 22.5 | 5.4 | N.D. | 0 |
| HC (4) | F, 56y | N.D. | N.D. | N.D. | N.D. | 26.3 | 33.8 | N.D. | N.D. | 9.7 | 4.8 | 18.1 | 0 |
| HC (5) | M, 67y | N.D. | N.D. | N.D. | N.D. | 63.8 | 28.8 | N.D. | N.D. | 13.6 | 6.8 | 11.2 | 0 |
| HC (6) | M, 49y | N.D. | N.D. | N.D. | N.D. | 10.1 | 19 | N.D. | N.D. | 9.5 | 2.1 | 7.4 | 0 |
| HC (7) | F, 36y | N.D. | N.D. | N.D. | N.D. | 40.6 | 36.5 | N.D. | N.D. | 15.7 | 6.1 | 5.1 | 0 |
| HC (8) | M, 48y | N.D. | N.D. | N.D. | N.D. | 10 | 16.9 | N.D. | N.D. | 10.9 | 6.4 | 7.4 | 0 |
| HC (9) | F, 41y | N.D. | N.D. | N.D. | N.D. | 18.8 | 22 | 84.8 | 15.1 | 11.1 | 2.9 | 12.7 | 0 |
| HC (10) | F, 50y | N.D. | N.D. | N.D. | N.D. | 9.6 | 15.9 | 68.9 | 13.5 | 10 | 2.8 | 1.2 | 0 |
| HC (11) | M, 53y | N.D. | N.D. | N.D. | N.D. | 72.6 | 22.6 | N.D. | N.D. | 19.9 | 9.7 | 2.8 | 0 |
| HC (12) | M, 65y | N.D. | N.D. | N.D. | N.D. | 36.2 | 35.3 | 66.4 | 32.2 | 21.9 | 9.7 | 33.2 | 0 |
| HC (13) | M, 44y | N.D. | N.D. | N.D. | N.D. | 46.1 | 33.6 | 99.5 | 89.9 | 14 | 6.5 | 2.1 | 0 |
| HC (14) | F, 56y | N.D. | N.D. | N.D. | N.D. | 12.6 | 14.6 | 83 | 46.2 | 3.9 | 1.5 | 0.9 | 0 |
| HC (15) | M, 64y | N.D. | N.D. | N.D. | N.D. | 41.6 | 20 | 70.8 | 42.4 | 9.7 | 5.4 | 2 | 0 |
| HC (16) | F, 55y | N.D. | N.D. | N.D. | N.D. | 11 | 17.6 | 93.8 | 27.1 | 8.3 | 4.2 | 4.9 | 0 |
| HC (17) | F, 41y | N.D. | N.D. | N.D. | N.D. | 13.7 | 20.4 | N.D. | N.D. | 11.3 | 5.4 | 8.1 | 0 |
| HC (18) | F, 31y | N.D. | N.D. | N.D. | N.D. | 26.2 | 39.9 | N.D. | N.D. | 2.4 | 1 | 3.9 | 0 |

PBC, primary biliary cholangitis; HC, healthy control; AMA-M2, anti-mitochondrial antibody subtype M2; ALT, alanine aminotransferase; AST, aspartate aminotransferase; ALP, alkaline phosphatase; GGT, gamma-glutamyl transferase; TBA, total bile salts; TBIL, total bilirubin; DBIL, direct bilirubin; AKR1B10, aldo-keto reductase family 1 member B10; IHC, immunohistochemistry; M, male; F, female. N.D., not detect. “-” denotes negative; “+” denotes positive.

**Supplementary Table 2. Screening DEGs in PBC by bioinformatic analysis from GEO datasets**

| **DEGs** | **Gene names** |
| --- | --- |
| Up-regulated | CXCL9, CXCL10, CXCL11, AKR1B10, UBD, FNDC4, FOXP3, CCL9, PEG10, FADS2, IGHD, IGHA1, IGLJ3, ACSL4, OLFM2, IGHM, IGHG1, MMP9, GPRC5B, LOXL4, FAM111B, IFI6 |
| Down-regulated | LY6G5B, LDLR, GPR21, WEE1, MIR30E, SNORD50A, GADD45G, SLED1, PHLDA1, MIR21, EGR1, FOS |

DEGs, differentially expressed genes; PBC, primary biliary cholangitis; GEO, gene expression omnibus.

**Supplementary Table 3. The results of GO analysis of DEGs**

| **Category** | **Term** | **Count** | **P value** | **Q value** |
| --- | --- | --- | --- | --- |
| BP | Positive regulation of lymphocyte activation | 7 | 1.46E-06 | 5.48E-04 |
| BP | Adaptive immune response based on somatic recombination of immune receptors built from immunoglobulin superfamily domains | 7 | 1.85E-06 | 5.48E-04 |
| BP | Humoral immune response | 7 | 2.09E-06 | 5.48E-04 |
| BP | Antimicrobial humoral response | 5 | 2.74E-06 | 5.48E-04 |
| BP | Positive regulation of leukocyte activation | 7 | 3.42E-06 | 5.48E-04 |
| BP | Positive regulation of cell activation | 7 | 4.34E-06 | 5.80E-04 |
| BP | Phagocytosis, recognition | 4 | 1.26E-05 | 1.18E-03 |
| BP | Chemokine-mediated signaling pathway | 4 | 1.26E-05 | 1.18E-03 |
| BP | Regulation of B cell activation | 5 | 1.53E-05 | 1.18E-03 |
| BP | Positive regulation of CD4-positive, alpha-beta T cell differentiation | 3 | 1.58E-05 | 1.18E-03 |
| CC | External side of plasma membrane | 8 | 2.68E-07 | 1.16E-05 |
| CC | Immunoglobulin complex, circulating | 4 | 6.97E-06 | 1.50E-04 |
| CC | Blood microparticle | 4 | 9.60E-05 | 1.38E-03 |
| CC | Immunoglobulin complex | 4 | 1.39E-04 | 1.50E-03 |
| CC | Peroxisomal membrane | 2 | 4.33E-03 | 3.11E-02 |
| CC | Microbody membrane | 2 | 4.33E-03 | 3.11E-02 |
| CC | Peroxisome | 2 | 2.11E-02 | 8.89E-02 |
| CC | Microbody | 2 | 2.11E-02 | 8.89E-02 |
| CC | Low-density lipoprotein particle | 1 | 2.11E-02 | 8.89E-02 |
| CC | Extrinsic component of synaptic membrane | 1 | 2.11E-02 | 8.89E-02 |
| MF | Chemokine activity | 8 | 1.34E-06 | 7.18E-05 |
| MF | CXCR chemokine receptor binding | 4 | 3.50E-06 | 9.40E-05 |
| MF | Chemokine receptor binding | 4 | 5.65E-06 | 1.01E-04 |
| MF | Immunoglobulin receptor binding | 4 | 9.63E-06 | 1.29E-04 |
| MF | G protein-coupled receptor binding | 2 | 1.21E-04 | 1.30E-03 |
| MF | Antigen binding | 2 | 1.76E-04 | 1.57E-03 |
| MF | Glycosaminoglycan binding | 2 | 5.99E-04 | 4.22E-03 |
| MF | Cytokine activity | 2 | 6.28E-04 | 4.22E-03 |
| MF | Histone acetyltransferase binding | 1 | 1.02E-03 | 5.74E-03 |
| MF | Cytokine receptor binding | 1 | 1.07E-03 | 5.74E-03 |

GO, Gene Ontology; DEGs, differentially expressed genes; BP, biological process; MF, molecular function; CC, cellular component.

**Supplementary Table 4. The results of KEGG analysis of DEGs**

| **Category** | **Term** | **Count** | **P value** | **Q value** |
| --- | --- | --- | --- | --- |
| KEGG | Viral protein interaction with cytokine and cytokine receptor | 4 | 3.53E-05 | 1.75E-03 |
| KEGG | Toll-like receptor signaling pathway | 4 | 4.12E-05 | 1.75E-03 |
| KEGG | Chemokine signaling pathway | 4 | 4.42E-04 | 1.26E-02 |
| KEGG | Cytokine-cytokine receptor interaction | 4 | 2.20E-03 | 4.69E-02 |
| KEGG | Fatty acid metabolism | 2 | 5.46E-03 | 9.31E-02 |
| KEGG | Human T-cell leukemia virus 1 infection | 3 | 8.69E-03 | 1.13E-01 |
| KEGG | PPAR signaling pathway | 2 | 9.30E-03 | 1.13E-01 |
| KEGG | Colorectal cancer | 2 | 1.21E-02 | 1.29E-01 |
| KEGG | IL-17 signaling pathway | 2 | 1.43E-02 | 1.31E-01 |
| KEGG | NF-kappa B signaling pathway | 2 | 1.74E-02 | 1.31E-01 |

KEGG, Kyoto encyclopedia of genes and genomes; DEGs, differentially expressed genes.

**Supplementary Table 5. The results of KEGG analysis of high-AKR1B10 expression group in PBC**

| **Category** | **Term** | **NES** | **P value** | **Q value** |
| --- | --- | --- | --- | --- |
| KEGG | Cell cycle | 2.47 | 0 | 0 |
| KEGG | P53 signaling pathway | 2.14 | 0 | 6.00E-04 |
| KEGG | Ecm receptor interaction | 2.09 | 0 | 2.00E-03 |
| KEGG | Small cell lung cancer | 1.95 | 0 | 7.57E-03 |
| KEGG | Focal adhesion | 1.81 | 0 | 4.36E-02 |
| KEGG | Chronic myeloid leukemia | 1.81 | 2.03E-03 | 3.71E-02 |
| KEGG | Pathways in cancer | 1.77 | 0 | 4.45E-02 |
| KEGG | Bladder cancer | 1.76 | 4.10E-03 | 4.58E-02 |
| KEGG | Melanoma | 1.69 | 1.98E-03 | 7.28E-02 |
| KEGG | Glioma | 1.69 | 3.93E-03 | 6.78E-02 |

KEGG, Kyoto encyclopedia of genes and genomes; AKR1B10, aldo-keto reductase family 1 member B10; PBC, primary biliary cholangitis.

**Supplementary Table 6. The results of GO analysis of high-AKR1B10 expression group in PBC**

| **Category** | **Term** | **NES** | **P value** | **Q value** |
| --- | --- | --- | --- | --- |
| BP | DNA unwinding involved in DNA replication | 2.28 | 0 | 2.57E-03 |
| BP | Mitotic cell cycle checkpoint signaling | 2.22 | 0 | 5.77E-03 |
| BP | Regulation of nuclear division | 2.17 | 0 | 9.00E-03 |
| BP | Regulation of mitotic nuclear division | 2.16 | 0 | 7.39E-03 |
| BP | Female meiotic nuclear division | 2.16 | 0 | 6.17E-03 |
| BP | DNA replication initiation | 2.15 | 0 | 6.63E-03 |
| BP | Microtubule cytoskeleton organization involved in mitosis | 2.07 | 0 | 2.45E-02 |
| BP | Mitotic G2 M transition checkpoint | 2.06 | 0 | 2.47E-02 |
| BP | Negative regulation of metaphase anaphase transition of cell cycle | 2.05 | 0 | 2.65E-02 |
| BP | Quinone metabolic process | 2.05 | 0 | 2.41E-02 |
| CC | Condensed chromosome centromeric region | 1.99 | 0 | 7.53E-02 |
| CC | Basement membrane | 1.94 | 0 | 6.92E-02 |
| CC | Cyclin dependent protein kinase holoenzyme complex | 1.89 | 1.92E-03 | 8.62E-02 |
| CC | Keratin filament | 1.88 | 2.07E-03 | 7.06E-02 |
| CC | Collagen containing extracellular matrix | 1.86 | 0 | 7.07E-02 |
| CC | Chromosome centromeric region | 1.85 | 0 | 7.02E-02 |
| CC | Chromosomal region | 1.85 | 0 | 6.04E-02 |
| CC | Mitotic spindle | 1.82 | 0 | 7.03E-02 |
| CC | Intermediate filament cytoskeleton | 1.82 | 0 | 6.33E-02 |
| CC | Condensed chromosome | 1.81 | 0 | 6.19E-02 |
| MF | Extracellular matrix structural constituent | 1.99 | 0 | 1.29E-01 |
| MF | DNA replication origin binding | 1.95 | 4.04E-03 | 1.11E-01 |
| MF | D threo aldose 1 dehydrogenase activity | 1.95 | 0 | 7.54E-02 |
| MF | Protein disulfide isomerase activity | 1.88 | 1.96E-03 | 1.30E-01 |
| MF | Threonine type peptidase activity | 1.88 | 4.15E-03 | 1.07E-01 |
| MF | NF kappab binding | 1.85 | 4.00E-03 | 1.25E-01 |
| MF | Alcohol dehydrogenase nadpplus activity | 1.80 | 0 | 1.74E-01 |
| MF | DNA secondary structure binding | 1.80 | 7.77E-03 | 1.58E-01 |
| MF | Cyclin dependent protein serine threonine kinase regulator activity | 1.76 | 7.83E-03 | 2.09E-01 |
| MF | Dynein complex binding | 1.75 | 1.20E-02 | 2.03E-01 |

GO, Gene Ontology; AKR1B10, aldo-keto reductase family 1 member B10; PBC, primary biliary cholangitis BP, biological process; MF, molecular function; CC, cellular component. NES, normalized enrichment score.
